# A Case of Spontaneous Tumor Lysis Syndrome in a Patient with Ovarian Cancer

**DOI:** 10.1155/2015/461870

**Published:** 2015-06-16

**Authors:** Kazuhiro Okamoto, Toshifumi Kinoshita, Miyuki Shimizu, Isoji Okura, Akinori Kawada, Koichi Mizobuchi, Midori Ando

**Affiliations:** ^1^Department of Obstetrics and Gynecology, Kagawa Rosai Hospital, 3-3-1 Zyotocho, Marugame-shi, Kagawa-ken 763-8502, Japan; ^2^Department of Pathology, Kagawa Rosai Hospital, 3-3-1 Zyotocho, Marugame-shi, Kagawa-ken 763-8502, Japan

## Abstract

Tumor lysis syndrome (TLS) is a potentially life-threating complication of tumors or chemotherapy treatment. TLS commonly occurs in hematological malignancies, but it is very rare in patients with a solid tumor. In cases of solid tumors, TLS usually occurs spontaneously and after the initiation of anticancer therapy, and it has a high mortality rate. We present the novel case of a 62-year-old woman with an ovarian tumor who spontaneously developed TLS. Surgical reduction of the tumor mass vastly improved her condition. She showed no sign of tumor recurrence 8 months after treatment. As TLS is life-threatening, successful treatments must be seriously considered.

## 1. Introduction

Tumor lysis syndrome (TLS) is characterized by the massive lysis of malignant cells and the rapid release of intracellular components into the systemic circulatory system. It requires immediate intervention and is sometimes lethal [1]. It is typically associated with hematopoietic malignancies, but it is rare in patients with solid tumors [2]. In cases of solid tumors, TLS usually occurs spontaneously or after antineoplastic treatment [3]. It has a mortality rate as high as 40% due to inadequate preventive treatment and delayed curative treatment [4]. There are few previous reports on TLS in patients with ovarian cancer, and all were associated with chemotherapy [5–10]. Herein, we are the first to report on a case of ovarian cancer with spontaneous TLS.

## 2. Case Presentation

**Figure 1:**
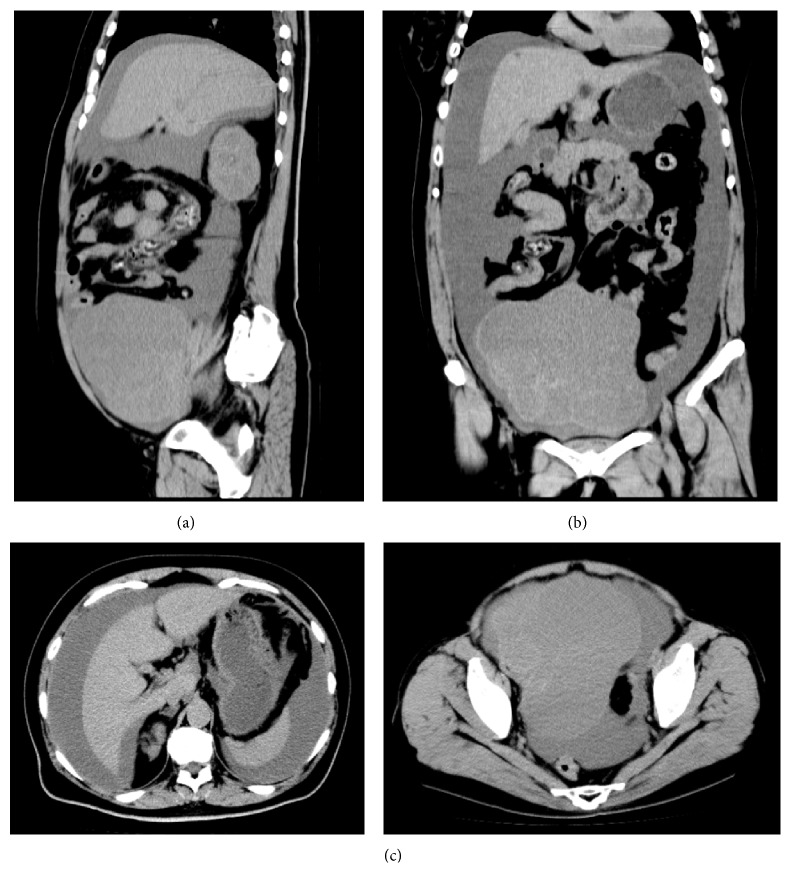
Abdominal plane computed tomography reveals a large pelvic mass with massive ascites that reach the liver and spleen. (a) Sagittal section; (b) frontal section; (c) coronal section.

The patient was a 62-year-old nonparous woman with no history of a gynecological disease. She had an enlarged abdomen during the 2 months preceding her visit to our hospital, and she had lower abdominal pain 2 days before her visit. She was transported to our hospital by ambulance and complained of disabling back pain. An abdominal examination revealed a palpable mass in the right lateral region of her abdomen. Transabdominal echography and computed tomography revealed a multilocular-solid tumor (>20 cm in diameter) in her pelvis, and massive ascites extended into the liver and spleen (Figure 1). Blood analysis showed hyperuricemia, hyperkalemia, hyperphosphatemia, and increased levels of plasma creatinine (Table 1). These findings fulfilled the diagnostic criteria of TLS.

**Figure 2:**
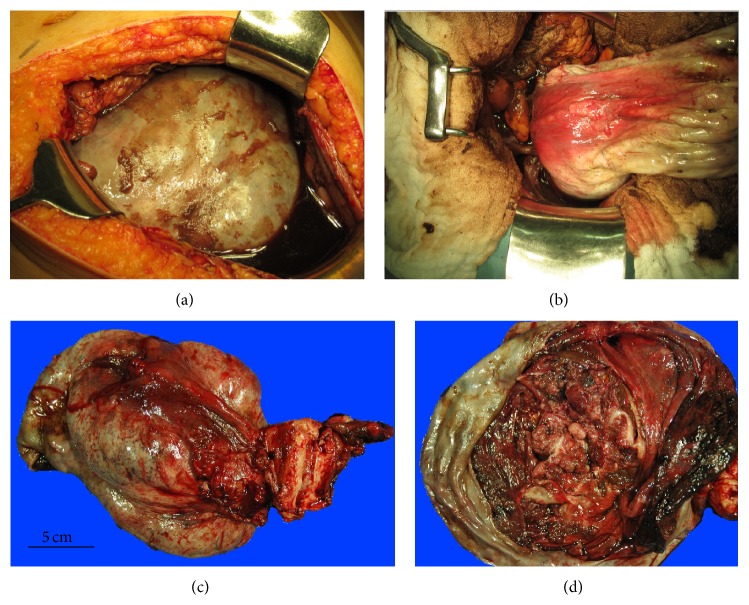
Operative findings. (a) The right ovary is markedly enlarged and contains a carcinoma. (b) The large ovarian tumor is removed via a vertical midline incision after abdominal drainage is performed. (c) The excised tumor mass is 20 cm in diameter. The uterus and left ovary are macroscopically normal. (d) The tumor mass contains septations and papillary solid lesions.

Following admission, the patient had anuria that responded poorly to aggressive fluid administration. We thought that her presentation could not be explained only by fluid shifts. Two days after admission, laboratory data indicated that her condition had worsened. Her respiratory status declined, and she was placed on a respirator. She also received a vasopressor to maintain her blood pressure. We decided to surgically reduce the amount of the tumor mass and to drain her abdomen in order to improve her condition. Abdominal bilateral salpingo-oophorectomy, hysterectomy, and abdominal drainage were performed (Figure 2).

We made a midline skin excision extending from the symphysis pubis to the right side of the umbilicus. The abdominal cavity was adequately exteriorized, and 3,300 mL of bloody, viscous fluid was suctioned. The large mass in the right ovary was extracted with the uterus and left ovary, which were macroscopically normal. The abdominal cavity was washed with saline to remove tumor tissue that adhered to the thickened, hyperemic peritonea. A drainage tube was placed in the upper abdomen, and the abdomen was closed. The patient received intensive care postoperatively, and her condition improved. The expression levels of biochemical markers returned to normal.

**Figure 3:**
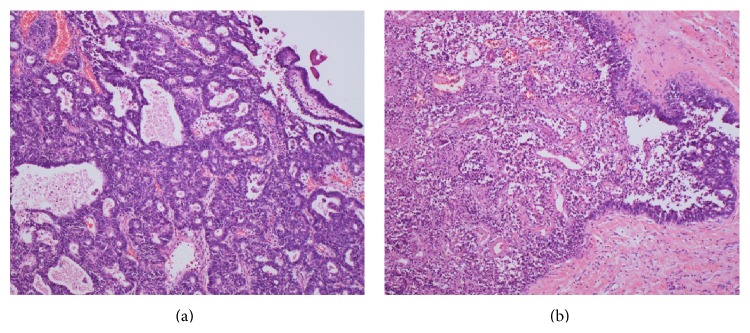
Pathological findings. (a) A grade 1 endometrioid adenocarcinoma is characterized by glandular patterns resembling those of the endometrium. (b) Extensive necrosis of the endometrioid carcinoma (left side). (a-b) Hematoxylin and eosin staining (magnification, ×100).

Pathologic examination of the neoplasm from the right ovary showed a well-differentiated (grade 1) endometrioid adenocarcinoma with extensive necrosis and capsular rupture (Figure 3). Cytology of the ascitic fluid showed no malignant cells. There were no remarkable changes in the macroscopic appearance of the left ovary or uterus. The final surgical pathologic diagnosis was stage IC ovarian endometrioid adenocarcinoma.

Postoperatively, the patient received six cycles of combination chemotherapy, which consisted of docetaxel and carboplatin. No signs of recurrence were observed at the 8-month follow-up.

## 3. Discussion

TLS is a potentially life-threatening complication of malignancies. It results from massive cytolysis of malignant tumor cells, either spontaneous or chemotherapy-induced, and the release of intracellular components into the circulatory system. TLS is characterized by hyperuricemia, hyperphosphatemia, hyperkalemia, hypocalcemia, increased plasma creatinine levels, and other abnormalities that can lead to acute renal dysfunction, fatal cardiac arrhythmia, central nervous system toxicity, and death [1]. The most widely accepted diagnostic criteria of TLS are provided in the Cairo-Bishop definition of laboratory TLS [1]. In our case, these criteria were met. TLS typically occurs in hematological malignancies, especially in acute lymphocytic leukemia and Burkitt's lymphoma, but it rarely occurs in solid tumors [2].

Potential risk factors of TLS in cases with solid tumors include liver metastasis, high lactate dehydrogenase and uric acid levels, renal insufficiency, prior treatment with nephrotoxic drugs, infection, and dehydration [11]. Solid tumors that may spontaneously rupture typically have a rapid growth, high volume, or chemosensitivity [3]. Six cases of ovarian cancer complicated by TLS have been reported in the literature, and all were associated with chemotherapy [5–10]. Conversely, we report on a novel case of spontaneous TLS in a patient with ovarian cancer. In this case, the risk factor for TLS appeared to be the large volume of the tumor. During surgery, we found that the tumor had ruptured, and the pathologic examination revealed extensive necrosis of the tumor. The rapid growth rate of the tumor presumably accounted for its large volume.

Treatment of TLS consists of aggressive hydration, the correction of electrolyte disturbances, and uric acid-lowering therapy [3]. The mortality rate of TLS in cases with solid tumors is higher than in cases with nonsolid malignancies, and it is reportedly as high as 40% [4]. Greater consideration of preventive treatments for TLS and the prompt treatment of TLS may aid in decreasing this rate. In the present case, the patient's condition worsened despite continuous intensive care. She required a respirator for breathing and a vasopressor to maintain her blood pressure. Because we thought that death was imminent, we performed surgery to reduce the amount of the tumor mass and to drain the abdomen in order to improve her condition. Postoperatively, her condition improved. Complete cytoreductive surgery removed most of the lysed tumor, which may have largely contributed to her improved condition. When nonsurgical treatment of TLS elicits a poor response or no response, debulking surgery seems to be a viable option.

TLS is a relatively rare event in patients with solid cancers. Nevertheless, clinicians should remember that such patients may develop this lethal complication in response to chemotherapy or even spontaneously.

## Figures and Tables

**Table 1:** Patient's laboratory data on admission.

CBC		Blood chemistry	
WBC	14,300/*μ*L	BUN	47 mg/dL
RBC	451 × 10^4^/*μ*L	Cr	2.98 mg/dL
Hb	14.4 g/dL	UA	8.1 mg/dL
Ht	41.2%	Na	132 mEq/L
Platelet	32.9 × 10^4^/*μ*L	K	4.9 mEq/L
Serological test		Cl	91 mEq/L
CRP	22.5 mg/dL	P	8.7 mg/dL
Coagulation		Glucose	435 mg/dL
FDP	16.3 *μ*g/mL	CEA	32.9 mg/mL
D-dimer	6.9 *μ*g/mL	CA19-9	1,999 U/mL
		CA125	4,325.1 U/mL

CBC: complete blood count, WBC: white blood cell, and RBC: red blood cell.

Hb: hemoglobin, Ht: hematocrit.

CRP: C-reactive protein, FDP: fibrin/fibrinogen degradation products, BUN: blood urea nitrogen, Cr: creatinine, UA: urinary acid, Na: sodium, K: potassium, Cl: chlorine, P: phosphorus, CEA: carcinoembryonic antigen, CA19-9: carbohydrate antigen 19-9, and CA125: carbohydrate antigen 125.
